# Diversity and Evolution of Bacterial Twin Arginine Translocase Protein, TatC, Reveals a Protein Secretion System That Is Evolving to Fit Its Environmental Niche

**DOI:** 10.1371/journal.pone.0078742

**Published:** 2013-11-13

**Authors:** Domenico Simone, Denice C. Bay, Thorin Leach, Raymond J. Turner

**Affiliations:** 1 Institute of Biomembranes and Bioenergetics, National Research Council, Bari, Bari, Italy; 2 Department of Biological Sciences, University of Calgary, Calgary, Alberta, Canada; Centre National de la Recherche Scientifique, Aix-Marseille Université, France

## Abstract

**Background:**

The twin-arginine translocation (Tat) protein export system enables the transport of fully folded proteins across a membrane. This system is composed of two integral membrane proteins belonging to TatA and TatC protein families and in some systems a third component, TatB, a homolog of TatA. TatC participates in substrate protein recognition through its interaction with a twin arginine leader peptide sequence.

**Methodology/Principal Findings:**

The aim of this study was to explore TatC diversity, evolution and sequence conservation in bacteria to identify how TatC is evolving and diversifying in various bacterial phyla. Surveying bacterial genomes revealed that 77% of all species possess one or more *tatC* loci and half of these classes possessed only *tatC* and *tatA* genes. Phylogenetic analysis of diverse TatC homologues showed that they were primarily inherited but identified a small subset of taxonomically unrelated bacteria that exhibited evidence supporting lateral gene transfer within an ecological niche. Examination of bacilli *tatCd*/*tatCy* isoform operons identified a number of known and potentially new Tat substrate genes based on their frequent association to *tatC* loci. Evolutionary analysis of these Bacilli isoforms determined that TatCy was the progenitor of TatCd. A bacterial TatC consensus sequence was determined and highlighted conserved and variable regions within a three dimensional model of the *Escherichia coli* TatC protein. Comparative analysis between the TatC consensus sequence and Bacilli TatCd/y isoform consensus sequences revealed unique sites that may contribute to isoform substrate specificity or make TatA specific contacts. Synonymous to non-synonymous nucleotide substitution analyses of bacterial *tatC* homologues determined that *tatC* sequence variation differs dramatically between various classes and suggests TatC specialization in these species.

**Conclusions/Significance:**

TatC proteins appear to be diversifying within particular bacterial classes and its specialization may be driven by the substrates it transports and the environment of its host.

## Introduction

The translocation and secretion of folded proteins often containing complex cofactors across the plasma membrane in archaea, bacteria and plant chloroplasts is accomplished by the twin arginine translocase (Tat) system (as reviewed by [1]). This translocation system, originally referred to as the membrane targeting and transport (Mtt) system [2], transports a number of substrates that generally possess an amino (N) terminal signal sequence with a conserved characteristic amino acid SRRXFLK motif (where X is typically a polar residue) containing the eponymous twin arginine residues as its name implies (as reviewed by [3]). The substrates themselves are diverse in nature, where some are involved in virulence [4], and others include membrane proteins [5]. Other Tat substrates such as respiratory enzymes (dehydrogenases and oxidoreductases), incorporate metal containing cofactors involving Ni-Fe, iron- sulfur [6], [7], thiocyanate [8], copper sulphur [9], [10] and molybdenum [8] (as reviewed by [11]).

The Tat system is composed of multiple integral membrane protein components TatA to TatE. TatA, TatC, and in some cases TatB (depending on host phylum) are essential components for function [12], [13], [14], [15]. The TatA family of proteins (∼90 amino acids (a.a.)) are proposed to form the translocation pore of the translocase complex that can adopt variable sizes to facilitate the movement of the targeted substrate across the membrane [16], [17]. The TatB (∼170 a.a.) and TatC (∼240 a.a.) family protein components can form autonomous multimeric subunits and/or heteromultimers that recognize and bind to the twin-arginine signal sequence of the substrate [18], [19], [20], [21], [22], and potentially the substrate itself [15], to assist TatA pore formation. The roles of other Tat components, TatD and TatE are less clear, as these components are only present in particular species and classes (as reviewed by [1]). Some researchers have proposed that TatD functions as a quality control component for system substrates [23], whereas others support the hypothesis that this protein is entirely unrelated to Tat mediated export [24]. TatE appears to be a functional duplication of TatA and may participate in transport pore formation [25], [26].

Of all the Tat system components, TatC has been extensively characterized in *Escherichia coli* (as reviewed by [1]). A recent high resolution X-ray diffraction crystal structure has been determined for *Aquifex aeolicus* TatC (4B4A) [27], and provides insight towards mechanisms and confirms that TatC spans the membrane as a six transmembrane α-helix protein [28]. Prior to the *A. aeolicus* TatC study, insight into TatC structure and function was obtained from a variety of *E. coli* TatABC experiments that involved amino acid residue substitutions [12], [29], [30], [31], Tat component gene deletions [32], [33], cross linking analyses [30], [34], and secretion experiments [35]. This information has contributed to understanding the involvement of many conserved amino acid residues found throughout the TatC sequence, and identified regions and residues that interact with other Tat components and substrate signal peptide sequences. These interactions emphasized the importance of the N- terminus and cytosolic facing loops and transmembrane (TM) regions of TatC that bind and recognize the twin arginine leader sequence [36]. Other studies of TatC identified that the periplasmic facing loops and TM regions participate in numerous TatA, TatB and TatC contacts [31], [34].

In general, most organisms possess a single core operon *tatABC* system, but multiple Tat systems can reside within a single host genome (as reviewed by [1]; Tables 1 and 2). In addition to studies involving the *E. coli* TatABC system, isoforms TatCd and TatCy present in *Bacillus subtilis* have also been examined [37]. In *Bacillus subtilis*, two distinct Tat systems are present, TatAd-TatCd and TatAy-TatCy, where each is responsible for the secretion of particular substrates, depending on phosphate availability [38], salinity [39], [40] and the relative abundance of TatC isoforms present in the plasma membrane [41]. In particular, PhoD is specifically secreted by the TatAdCd system and YwbN (also referred to as EfeB), YkuE and QcrA are specifically secreted by the TatAyCy system [42], [43], [44]. The TatAyCy translocase was also recently shown to play a role in biofilm formation by *B. subtilis* [42]. Thus, the presence of multiple Tat systems within a single organism may be important to facilitate the secretion of substrates under a given environmental condition.

**Table 1:** A summary of Tat translocase subunits (A/E, B, C) present in each bacterial class surveyed for this study and the most common arrangement of these genes in *tat* operons.

Bacterial Class	Total species identified	Total number of genes identified in class			*tat* operon§ arrangement
		*tatA/E*	*tatB*	*tatC*	
Actinobacteria	118	152	78	118	AC ↔ (B/A)
Bacilli	51	57	1	58	AC ↔ (AC)
Clostridia	29	59	7	29	AC
Deinococci	14	35	72	15	AC
α-proteobacteria	129	148	121	131	ABC or C↔A
β-proteobacteria	76	91	74	80	ABC
γ-proteobacteria	166	232	146	170	A^2^BC(D)
δ-proteobacteria	37	76	23	39	(A/B)C ↔ (A)
ε-proteobacteria	24	21	9	24	BC ↔ A
Chloroflexi	14	361	9	15	A^2^BC
Planctomycetia/Phycisphaerae	5	9	0	5	C ↔ A^2^
Chlamydiae	2	2	0	2	AC
Aquificae	9	17	8	9	A^2^BC
Bacteroidetes	44	61	3	45	AC ↔ (A/B)
Cyanobacteria	15	22	3	17	C ↔ A2
Deferribacteres	4	8	3	4	BC ↔ A2
Fusobacteria	2	2	1	2	C(B)A
Gemmatimonadetes	1	1	0	1	C ↔ A
Chlorobia/Ignavibacteria	11	11	0	11	C ↔ A
Negativicutes	5	5	0	5	AC
Nitrospira	3	6	0	3	C ↔ A^2^
Acidobacteria/Solibacteres	6	9	3	6	(B)C ↔ A^2^
Sphingobacteria	8	8	0	8	C ↔ A
Spirochaetes	4	4	0	4	AC
Thermodesulfobacteria	2	2	0	2	AC
Verrucomicrobia	4	8	0	4	C ↔ A^2^

§ *tat* genes are listed as single letters A to C and combinations written together indicate their presence at the same *tat* operon locus. Bidirectional ↔ arrows indicate that the Tat component genes are not found in the same operon (separate loci). *tat* genes (A, B or C) found in parentheses indicate an optional occurrence in the *tatC* locus.

**Table 2:** A summary of bacterial genomes in possession of more than one *tatC* locus identified from this survey.

Bacterial class	Genus and species	Number of genomes surveyed	Number of TatC loci/genome
Actinobacteria	*Corynebacterium variabile*	1	2
	*Microbacterium testaceum*	1	2
	*Nakamurella multipartite*	1	2
	*Streptomyces hygroscopicus*	1	2
	*Xylanimonas cellulosilytica*	1	2
Bacilli	*Bacillus amyloliquefaciens*	8	2
	*Bacillus atrophaeus*	1	2
	*Bacillus halodurans*	1	2
	*Bacillus licheniformis*	2	2
	*Bacillus megaterium*	3	2
	*Bacillus sp. JS*	1	2
	*Bacillus subtilis*	5	2
	*Geobacillus sp. Y4.1MC1*	1	2
	*Geobacillus thermoglucosidasius*	1	2
	*Lysinibacillus sphaericus*	1	2
	*Paenibacillus mucilaginosus*	3	2
	*Paenibacillus sp. JDR-2*	1	3
	*Paenibacillus sp. Y412MC10*	1	4
Clostridia	*Sulfobacillus acidophilus*	2	2
	*Thermodesulfobium narugense*	1	2
β-proteobacteria	*Methylobacillus flagellates*	1	2
	*Methylotenera mobilis*	1	2
δ-proteobacteria	*Desulfotalea psychrophila*	1	2
	*Geobacter lovleyi*	1	2
	*Desulfurivibrio alkaliphilus*	1	2
γ-proteobacteria	*Acinetobacter calcoaceticus*	1	2
	*Acinetobacter oleivorans*	1	2
	*Acinetobacter sp. ADP1*	1	2
	*Cellvibrio japonicas*	1	2
	*Colwellia psychrerythraea*	1	2
	*Glaciecola sp. 4H-3-7+YE-5*	1	2
	*Pseudoalteromonas atlantica*	1	2
	*Pseudomonas putida*	6	2.16*
	*Psychrobacter sp. Prwf-1*	1	2
Cytophagia	*Cytophaga hutchinsonii*	1	2
Nitrospira	*Candidatus Nitrospira defluvii*	1	2
Solibacteres	*Candidatus Solibacter usitatus*	1	2
Thermodesulfobacteria	*Thermodesulfatator indicus*	1	3
	*Thermodesulfobacterium sp. OPB45*	1	3

* The number of *tatC* loci retrieved was two loci in five genomes surveyed and three loci in one genome out of a total of six available completed *P. putida* genomes.

The focus of this study was to closely examine the bacterial TatC protein family using bioinformatics approaches to explore its diversity and evolution and to identify residues and regions important for TatC structure, function and interactions to other Tat components. This examination expands upon previous bioinformatics analyses [45] by surveying a larger number of sequenced bacterial genomes (4373), through generating an extensive taxonomically diverse bacterial TatC protein and nucleotide dataset. A combination of protein sequence phylogenetic analyses and nucleotide sequence synonymous to non-synonymous substitution rates (dS/dN) were used to evaluate TatC evolution and the pressures driving their divergence within particular bacterial phylum and classes. This examination also included an in depth examination of Bacilli TatCd/TatCy isoforms evolution by phylogenetic analysis in addition to *tatC* operon surveying to identify candidate genes that contribute to translocase divergence. The results identified that the majority of TatC sequences follow taxonomic inheritance in both Gram- negative and Gram- positive bacteria similar to previous phylogenetic analyses [45]. However, this extensive TatC sequence analysis identified a smaller secondary group of taxonomically unrelated bacteria that inherit *tatC* sequences through horizontal gene transfer from a shared common environmental niche. Examination of Bacilli species with single and multiple TatC isoforms revealed that TatCy appears to be the progenitor of TatCd. It also appears that TatCy in particular is gained much more frequently by lateral gene transfer events rather than gene duplications suggesting that horizontal gene transfer influences isoform substrate divergence. Finally, a comparative analysis between the overall bacterial TatC amino acid sequence conservation and Bacilli TatCd/TatCy amino acid sequence alignments revealed conserved and variable residues and regions within TatC that likely play important roles for the function of Tat translocase.

## Methods

### TatC Protein and Nucleotide Sequence Dataset Collection

**Figure 1:**
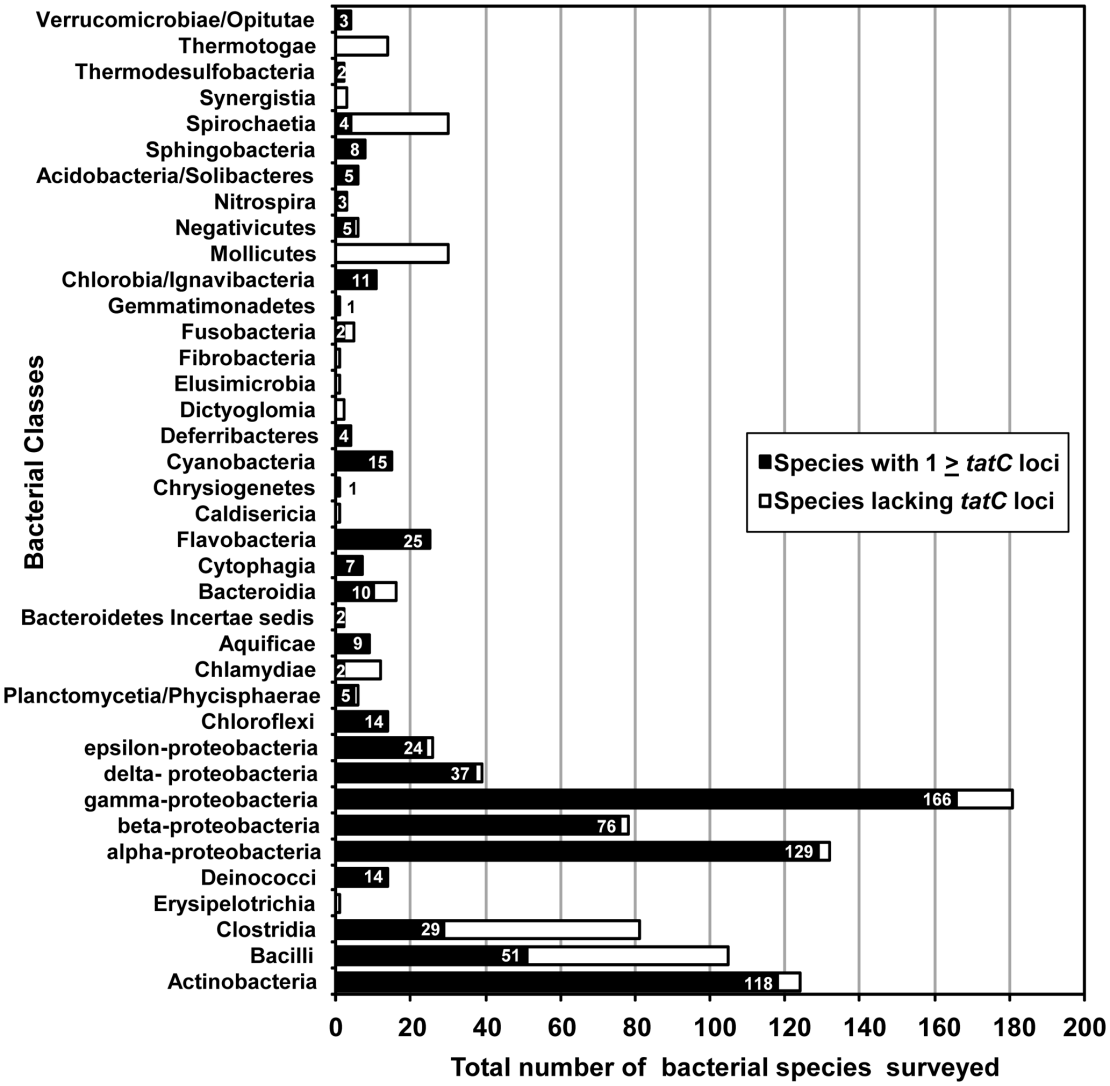
The bar plot distribution of *tatC* sequences in bacterial genomes. Bacterial species surveyed from 44 different classes are provided on the y-axis and the total number of species genomes surveyed is provided on the x-axis. Bars indicate the total number of surveyed species with one or more *tatC* sequences (black) and surveyed species that lacked *tatC* homologues (unfilled) within a given bacterial class. Numbers provided in each bar plot indicate the total number of species having one or more *tatC* sequence is reported for each bacterial class. Bacterial species with more than one *tatC* encoded within its genome is summarized on Table 2. At the time of the survey (July 2012) a total of 1018 bacterial species from 44 classes were examined and 782 bacterial species genomes in total possessed one or more *tatC* locus. Data for this distribution is provided in Figure S1.

The TatC protein and nucleotide sequence datasets analyzed in this study were collected from completed sequences bacterial genome collection on the National Center of Biotechnology Information (NCBI) website (http://www.ncbi.nlm.nih.gov/) with the tBLASTn available through BLAST suite programs from NCBI Microbial Genome Resources [46]. TatC proteins were identified from these genomes searches using TatC amino acid sequences from *Escherichia coli* (Swiss-Prot: P69424) and *Bacillus subtilis* (TatCd, Swiss-Prot: P42252 and TatCy, Swiss-Prot: O05523) as query sequences to search for *tatC* homologues with an E-value cut-off of ≤10^−4^. As of June 2012, the number of available completed bacterial genome sequences was 4373 (2422 are plasmids and 1951 are chromosomes) and corresponded to a total of 1018 bacterial species. tBLASTn searches resulted in a total of 1415 genomic *tatC* loci out of a total 782 bacterial species (77% of the total species surveyed). A list of all TatC sequences identified in this study is provided in Table S1 according to its corresponding NCBI accession number. In addition to *tatC* sequences, a summary of Tat translocase components (TatA/E, TatB and TatC) identified in *tat* operons for each bacterial class surveyed in this study is also provided and listed in Table 1. A summary of bacterial TatC distribution is provided in Figure 1 and Table 2. All identified TatC sequence homologues were chromosomally encoded except for *Ilyobacter polytropus*, from the order Fusobacteriales, which had a plasmid encoded *tatC*.

### Multiple Sequence Alignments of TatC Datasets

Multiple sequence alignments of TatC amino acid sequence datasets used for phylogenetic and synonymous/non-synonymous analyses and consensus sequence determinations were performed using three different programs, Multiple Sequence Comparison by Log-Expectation (MUSCLE), Multiple Alignment using Fast Fourier Transform (MAFFT) and hidden markov model alignment (hmmalign) software of the HMMER3 v3.1 package [47], [48]. The hmmalign software was applied by aligning each of the 1415 sequences in the TatC dataset against a Hidden Markov Model (HMM) alignment profile of the TatC protein family available at Pfam database (http://pfam.sanger.ac.uk/) with accession PF00902. The application of the hmmalign software aided in the multiple alignment of distantly related TatC homologue sequences. Non-confidently aligned residue columns were omitted from phylogenetic analyses and synonymous/non-synonymous nucleotide substitution analyses. Poorly aligned residues within amino- and carboxyl- termini and within loop regions were generally removed from multiple alignments due to low conservation in these regions.

To selectively represent non-redundant taxonomically diverse TatC sequences for phylogenetic and synonymous/non-synonymous analyses the 1415 TatC dataset was analyzed using the web based BLASTClust program (http://toolkit.tuebingen.mpg.de/blastclust) to aid in the selection of 232 unique, taxonomically diverse TatC sequences that represented all bacterial phyla surveyed. Multiple alignment editing was performed with custom Python scripts and with the multiple alignment editing program Jalview [49]. Jalview was used to calculate amino acid sequence percentage identity (% identity) values that equated to the consensus residue at each position (Figure S1). Jalview was also used to generate and edit a Bacilli TatC multiple alignment that consisted of 57 sequences, where 31 sequences were Bacilli TatCd and 26 sequences were Bacilli TatCy. Both Bacilli TatCd and TatCy multiple sub-alignment datasets were used to calculate amino acid percentage identity values at each position. Two separate TatC datasets, where Bacilli TatCd/TatCy isoforms were either included or excluded were also aligned to determine if percentage identity values differed in the overall TatC residue consensus. No significant differences were detectable between TatC consensus with and without Bacilli TatCd/Cy isoforms and only the TatC consensus including Bacilli isoforms was used in this study.

TatC transmembrane domain predictions for TatC sequences were performed using local installations of programs transmembrane hidden markov model (TMHMM) [50], dense alignment surface (DAS) [51] and membrane protein structure and topology (MEMSAT) [52] where the overlapping transmembrane domain predicted from all three programs were used to determine each of the six predicted transmembrane regions in the protein and all of these regions are highlighted in blue in Figure S1.

### Phylogenetic Analysis of Bacterial TatC Proteins

**Figure 2:**
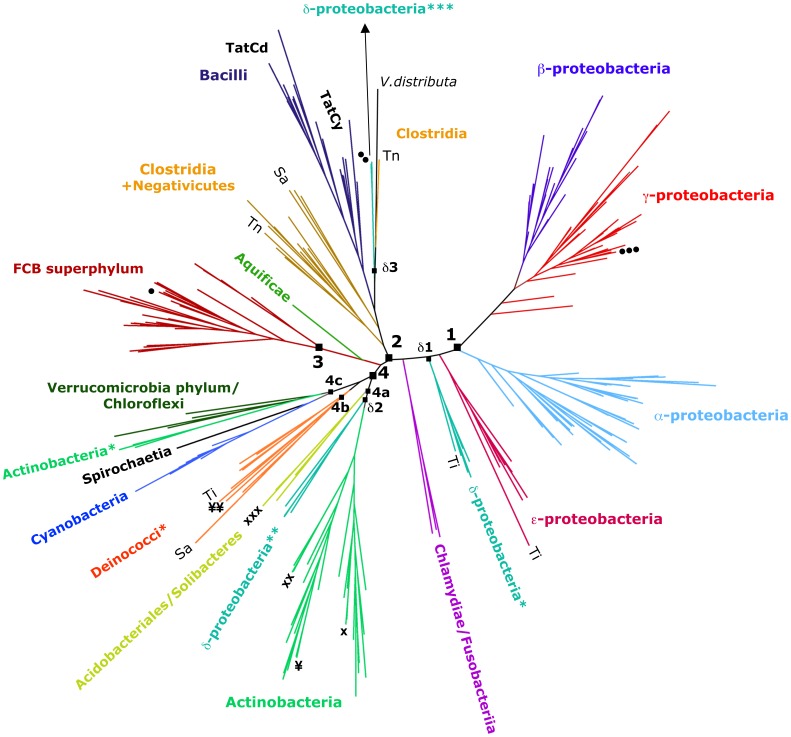
Phylogenetic analysis of TatC protein sequences from diverse bacterial taxa. The unrooted phylogenetic tree is based on a Bayesian analysis of 232*Vulcanisaeta distributa* (AC: YP_003902595.1), is labelled as “*V.distributa*”. Supporting values at each node and individual labels, genus and species names, of all TatC sequences are not shown in this tree for clarity but are provided in the rooted version of the tree in Figure S2. Taxa are described according to their bacterial class, except for classes Verrucomicrobiae and Opitutae (collectively referred to as “Verrucomicrobia phylum”) and classes Chlorobia, Ignavibacteria, Cytophagia, Bacteroidia, Flavobacteria and Sphingobacteria (collectively referred to as “Bacteroidetes/Chlorobi phyla”). Taxa marked with asterisks are detailed as follows: Actinobacteria* includes TatC homologues from class Coriobacteria; Deinococci* includes TatC homologues from classes Deinococci, Thermodesulfobacteria, Nitrospira and Rubrobacteriia; δ-proteobacteria (*) includes TatC homologues from δ-proteobacterial orders Desulfovibrionales and Desulfobacterales; δ-proteobacteria (**) includes TatC homologues from δ-proteobacterial order Myxococcales; δ-proteobacteria (***) includes TatC homologues from δ-proteobacterial order Desulfuromonadales. Symbols adjacent to taxa indicates multiple TatC copies from the same genomes discussed in the Results and Discussion section and the corresponding species are detailed as follows: *Cytophaga hutchinsonii* (•); *Geobacter lovely* (••); *Colwellia psychrerythraea* (•••); *Xylanimonas cellulosilytica* (x); *Streptomyces hygroscopicus* (xx); *Candidatus Solibacter usitatus* (xxx); *Nakamurella multipartite* (¥); *Candidatus Nitrospira defluvii* (¥¥); *Sulfobacillus acidophilus* (Sa); *Thermodesulfobium narugense* (Tn); *Thermodesulfatator indicus* (Ti). Labeled nodes are discussed in the Results and Discussion section.

**Figure 3:**
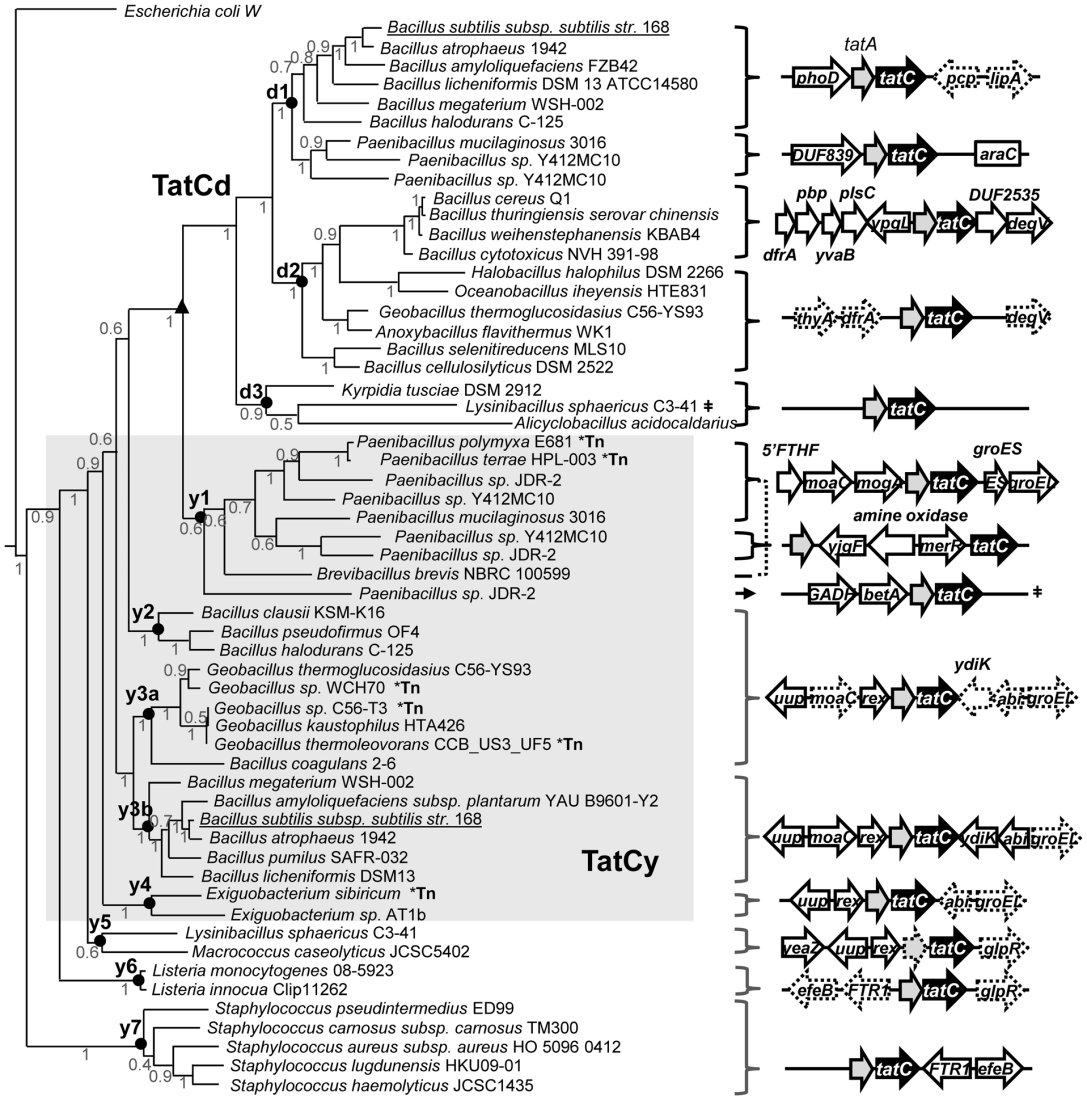
Phylogenetic analysis of bacilli TatCd and TatCy proteins and *tatC* operon conservation. Bayesian analysis was performed to generate the dendrogram provided on the left hand side of the panel and shows the homology between 57 representative bacilli TatC sequences including characterized *B. subtilis* TatCd and TatCy seed sequences (underlined). Nodes of importance are indicated by labelled black circles based on its relationship to either TatCd (d1–d3) or TatCy (y1, y2, y3a, y3b- y7). *Bacillus* species marked by an asterisk ‘Tn’ indicate that its corresponding *tatC* operon was adjacent to transposons or integron sequences. The arrow and box line diagrams shown on the right hand side of the dendrogram summarize *tatC* operon conservation for the indicated species. White filled arrows/boxes represent conserved genes located upstream and downstream from *tatA* (grey arrows) and *tatC* (black filled arrows). The direction of each arrow represents the open read frame direction relative to *tatC* and boxes represent genes present in either reading frame. Boxes and arrows with dotted lines indicate genes with moderate conservation 50–80% of *tatC* loci and solid lines indicate ≥80% gene conservation. The triangle symbol located on the dendrogram points out the division between the TatCd and TatCy clades. The symbol (≠) after *L. spaericus* indicates that it shares a similar *tatC* operon arrangement to that shown for sequences in the distal node of y1. Full names for abbreviated genes are listed as follows: alkaline phosphatase (*phoD*), pyrrolidone-carboxylate peptidase (*pcp*), domain of unknown function protein (DUF839), lipid esterase A (*lipA*), hypothetical lipid binding phosphotransferase (*degV*), domain of unknown function protein (DUF2535), arabinose family transcriptional regulator (*araC*), dihydrofolate reductase A (*dfrA*), thymidylate synthase A (*thyA*), periplasmic binding protein domain 3 (*pbp*), azoreductase (*yvaB*), 1-acyl-sn-glycerol 3-phosphate acyltransferase (*plsC*), 5-formyltetrahydrofolate cyclo ligase (5′FTHF), riboendonuclease liver perchloric acid-soluble protein (L-PSP) (*yjgF*), molybdenum cofactor biosynthesis protein C (*moaC*), molybdopterin biosynthesis protein (*mogA*/*moeA*), small and large subunit chaperones (*groES*/groEL), mercury resistant transcriptional regulator (*merR*), gluconate 2 dehydrogenase subunit 3 (GADH), gluconate 2 dehydrogenase flavoprotein (*betA*), ABC transporter ATP-binding protein- Elongation factor 3 (*uup*), redox sensing transcriptional repressor (*rex*), abortive infection CAAX protease protein (*abi*), iron dependent DyP-type peroxidase (*efeB/ywbN*), DUF4305 a 53 amino acid lipoprotein (*ydkI*), glycoprotein endopeptidase peptidase M22 (*yeaZ*), transcriptional regulator of sugar metabolism (*glpR*), high-affinity Fe^2+^/Pb^2+^ permease (FTR1/*efeU*).

Phylogenetic analysis of the 232 TatC protein sequence dataset was performed using Bayesian inference provided by MrBayes v3.2.1 software [53]. Additional phylogenetic analysis was also performed with this dataset using the Approximate Maximum Likelihood analysis method from FastTree software [54]. Maximum likelihood dendrograms showed similar branching patterns as Bayesian analysis but was not included herein based on superior confidence values at major nodes produced by Bayesian posterior probability (PP) values as compared to bootstrap calculations [55]. The conditions for Bayesian inference of the 232 TatC dataset were obtained using 17 million generations and 12 chains, setting a burn-in rate of 25% of the samples and by sampling the tree space every 500 generations. Model jumping between fixed-rate amino acid models was used to determine the most suitable substitution model. Our analysis favoured the Blosum62 substitution model [56]. Convergence of modelling was assessed using the standard deviation of the split frequencies and was determined to be 0.017 at the end of the analysis. Bayesian PP values were calculated via Markov chain Monte Carlo sampling (MCMC) methods to determine the confidence at all branch nodes and these values are provided in Figure S2. The Bayesian dendrogram of the 57 Bacilli protein alignment dataset was obtained with the same approach, but involved 3 million generations to reach convergence and the standard deviation of split frequencies was 0.004. The Bayesian dendrograms of the 232 TatC sequences is provided in Figure 2 as an unrooted tree and in rooted form in Figure S2 and the Bacilli TatCd and TatCy dendrogram is shown in Figure 3. The archaeal *Vulcanisaeta distributa* TatC protein sequence (YP_003902595.1) served as the outgroup for phylogenetic analysis (Figure 2 and Figure S2) and *E. coli* W strain TatC (YP_006175467.1) was the outgroup for Bacilli TatC analysis (Figure 3).

### Bacterial *tatC* Operon Analysis

Bacterial *tatC* operon analysis was determined by examining the first 10 open reading frames (both upstream and downstream) adjacent to the *tatC* locus/loci from annotated *tatC* sequence locus tags during genome surveys described above. In some cases, *tatA* and *tatB* loci were also sought out within these genomes to confirm legitimate absences from the TatC locus using NCBI tBLASTn searches with *E. coli* TatA (GenBank: YP_006126698) and TatB (GenBank: YP_006126699) as query sequences. The results of this analysis are presented in Table 1.

Examination of *tatC* loci present within Bacilli genomes (and for all bacteria in Table 1) was performed by retrieving the TatC locus tag (NCBI) for all 57 sequences in the Bacilli *tatC* dataset from its respectively annotated genome. Gene annotations corresponding to the first ten genes upstream and downstream from *tatC* were identified based on annotation. The frequency of occurrence for a particular gene at all *tatC* loci, and at either *tatCd* or *tatCy* loci was calculated by the number of times its gene name and/or by its enzyme name was identified from gene annotations. The frequency of gene occurrence (Freq) was determined by the equation [Freq = (n^gene^/n^locus^ ) x O*^tatC^*] where n^gene^ is the number of times a gene is identified at one of ten loci before or after the *tatC* locus, n^locus^ is the number of total loci examined, and O*^tatC^* is total number of *tatC* operons examined. The reading frame of all genes was also considered in the frequency of gene occurrence calculations as well as its proximity to the *tatC* locus. All of these parameters were used to generate the *tatC* operon diagrams shown in the right hand panel of Figure 3.

### Synonymous/Non-synonymous Nucleotide Substitution Analyses of *tatC*

**Figure 4:**
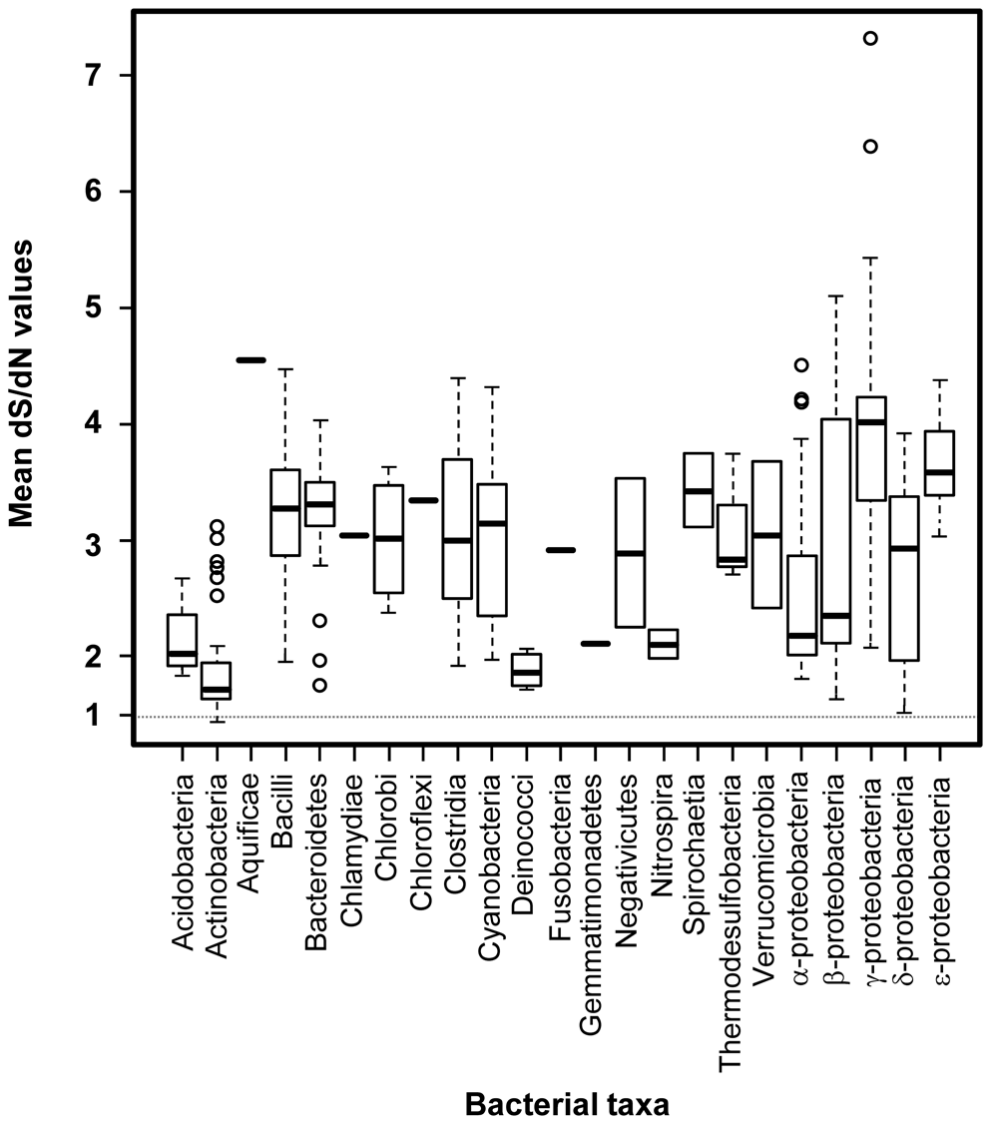
Boxplot distributions of mean synonymous to non-synonymous (dS/dN) changes in bacterial *tatC* homologues. Rectangle boxes represent the range of dS/dN values (y-axis) from the 75^th^ percentile (top of box) to the 25^th^ percentile (bottom of box) and a thick horizontal line within the box represents the median dS/dN value for all *tatC* species determined from each bacterial class (x- axis). Lines extending from the bottom of each box chart represents the range of values for the 25^th^ percentile –1.5*IQR (interquartile range, defined as the difference between the 75^th^ and the 25^th^ percentile) and the top lines represent the 75th percentile +1.5*IQR. Outliers are represented as unfilled circle dots. The dotted horizontal line at dS/dN value of 1 indicates the neutral selection cutoff. All bacterial taxa are described according to their classes on the x-axis with the exception of phyla Acidobacteria, Bacteroidetes, Chlorobi and Verrucomicrobia.

**Figure 5:**
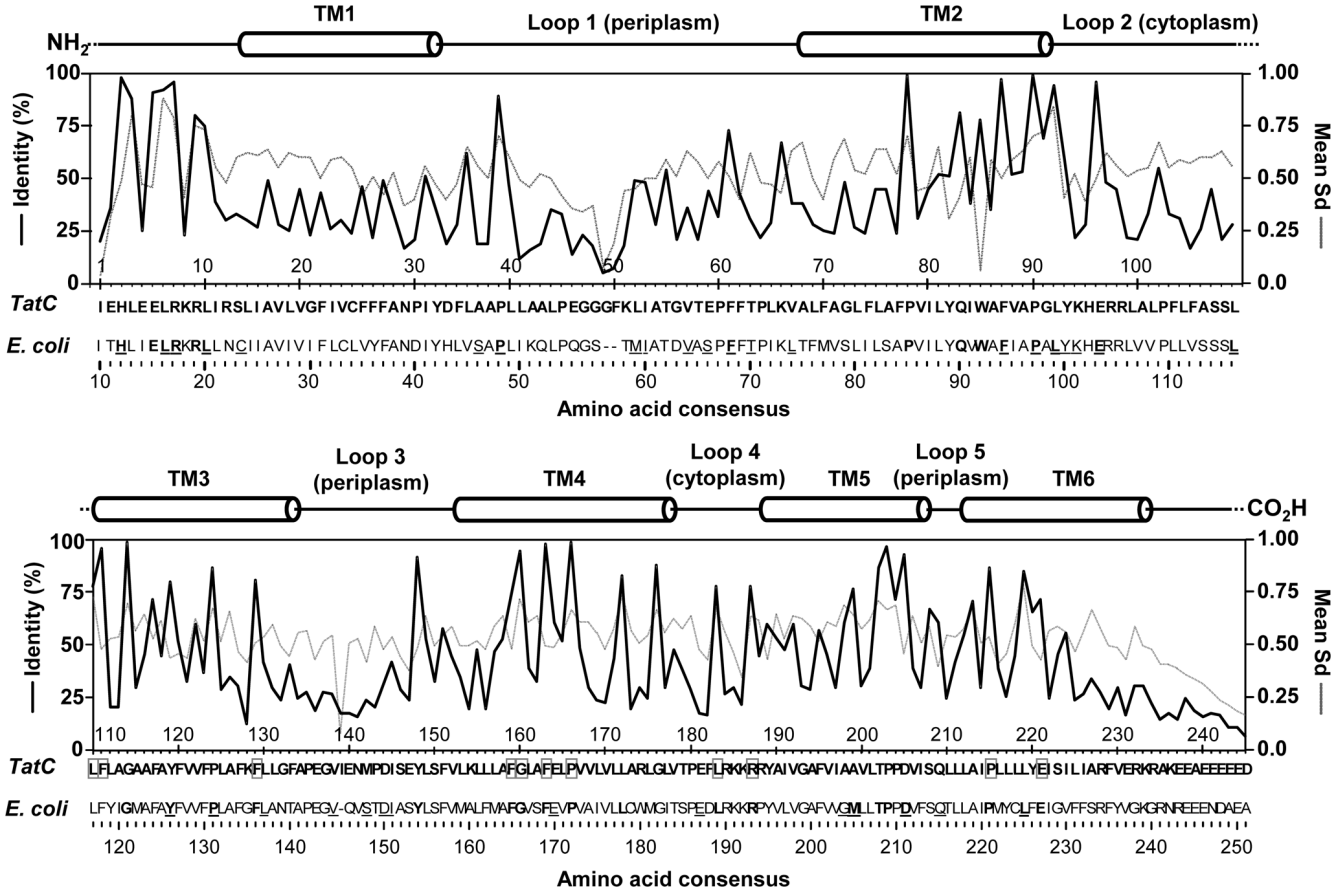
A summary of positional amino acid conservation in TatC proteins and its mean Synonymous substitution value (Sd) at each corresponding codon. Amino acid conservation (% identity; black line) and mean Sd (grey dotted line) values are shown as line graphs in both the top and bottom panels of each chart starting from the first amino acid position to the last alignable amino acid from the 232 TatC protein/*tatC* nucleotide dataset. The bottom x- axis shows each amino acid position (as single letter abbreviations), numbered according to their position in the overall alignment, determined from the multiple alignment of TatC proteins and its corresponding % identity (left y-axis) and mean Sd (right y-axis) values at each position. Residues listed below the TatC consensus sequence on the x-axis represent aligned *E. coli* TatC residues. Underlined amino acids in *E. coli* TatC residues indicate residues examined by site-directed mutagenesis that were shown to reduce or inactivate TatC substrate secretion [29], [30], [31], [34], [36]. Bolded letter indicate residues with equal to or greater than 75% identity. Residues outlined by grey boxes indicate residues with high % identity that have not been examined in previous studies (Table 3). A cartoon diagram representing TatC secondary structure elements is provided above each bar chart and indicates where loop regions (lines) and amino (NH_2_)/carboxyl (CO_2_H) termini (lines) and predicted α-helical TM strands (cylinders) correspond.

Synonymous to non-synonymous nucleotide substitution analyses were performed with synonymous non-synonymous analysis program (SNAP) package [57] available online (http://hcv.lanl.gov/content/sequence/SNAP/perlsnap.html). Generation of codon aligned *tatC* nucleotide datasets used for this analysis were performed with the program RevTrans [58] to obtain a multiple sequence codon alignment suitable for SNAP analysis. The results of SNAP analysis relative to the whole selected dataset of 232 *tatC* sequences are summarized in Figure 4 and Table S2. For each *tatC* sequence included in the analysis, a mean dS/dN value was calculated from all pairwise comparisons to other sequences in the same selected dataset. Pairwise dS/dN values could only be determined for 39% of all pairwise alignments of 232 *tatC* sequences included in this study since the remaining pairwise dS/dN values had pS values of 0.75 that prevented a reliable Jukes-Cantor transformation calculation of the dS value. Mean dS/dN values are reported in Table S2 and were used to calculate interquartile ranges (IQR) within each bacterial class as described in Figure 4 using the *boxplot* function as part of the R program version 2.15.2 (http://developer.r-project.org/). The number of observed synonymous nucleotide substitutions (Sd) at each codon was calculated using the Perl script *codons-xyplot.pl* available within the SNAP software package and the mean Sd values are summarized in Figure 5. Codon alignments for bacilli *tatCd* and *tatCy* were also analyzed using SNAP to determine pairwise dS/dN values for the entire protein in addition to Sd values for each codon in the multiple alignment resulting in mean Sd values.

### Structure Modelling of *E. coli* TatC Protein

**Figure 6:**
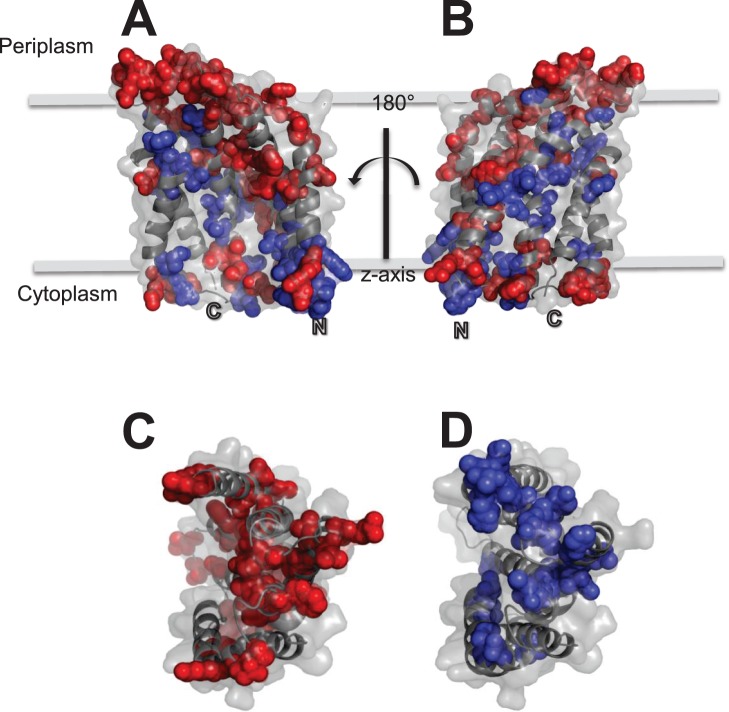
A structural model of *E. coli* TatC. In all panels, *E. coli* TatC proteins are shown as cartoon diagram where secondary structure ribbon diagrams are overlaid onto space filled structures (light grey). In all panels, amino acid residues are highlighted according to their conservation, where residues present at greater than 75% identity (blue) or less than 25% identity (red) based on the consensus data provided in Figure 5 are represented as coloured spheres according to their location in the TatC structure. Panels A to B show cartoon ribbon-space filled diagrams of the *E. coli* TatC protein within the membrane bilayer plane at 180° rotations from each other. Panels C and D show the TatC protein at a 90° rotation along the x-axis from the membrane plane shown in panels A-B, showing the cytoplasmic facing loops of the TatC structure. The models shown in all panels were built using the *Aquifex aeolicus* TatC structure pdb file (4B4A) [27] as described in the Methods section.

A homology model of *E. coli* TatC structure was generated from the recently published X-ray crystal structure of *Aquifex aeolicus* TatC [27] and shown in Figure 6. Before modelling was performed, the program T-coffee [59] was used to make a pairwise alignment between *E. coli* TatC (GenBank: YP_006175467.1) and *A. aeolicus* TatC (Swiss-Prot: O67305) protein sequence. This alignment was used to generate 10 models based on the *A. aeolicus* TatC crystal structure (PDB: 4B4A [27]), using the MODELLER program [60]. The best structure was selected based on lowest discreet optimized protein energy (DOPE) score, calculated by MODELLER, and the secondary structure and backbone angles of the model were further verified with the program PDBsum [61].

## Results and Discussion

### Distribution of *tatC* in Bacteria

Before beginning phylogenetic and sequence-based analyses of bacterial *tatC* homologues a survey of *tatC* distribution was performed to determine the extent of bacterial *tatC* possession within sequenced bacterial genomes. Based on the results of this survey, the occurrence of one or more *tatC* loci was found in 77% of all available completely sequenced bacterial genomes as shown in Figure 1 similar to the findings reported in a previous TatC study [45], [62]. In general, bacteria had one *tatC* gene/species on average based on this analysis. The arrangement of *tatC* within the *tat* operon varied greatly depending on each bacterial class surveyed in this study (Table 1). Surprisingly, TatAC systems were identified in 53% of all bacterial classes possessing *tatC* genes and in many of these classes *tatC* and *tatA* genes occurred at distantly separated loci within the genome suggesting that in these instances separated *tat* gene expression may be regulated under different conditions. *tatB* appeared to occur most often in Gram- negative classes and was infrequently found within Actinobacteria subclasses as a separate distant locus (Table 1). This may suggest that the requirement for *tatB* may be related to the type of substrates these systems transport. It is also noteworthy that the majority (89%) of species with *tatC* homologues were typically facultative anaerobes, obligate anaerobes and facultative aerobes. Very few species possessing Tat systems had an obligate aerobic physiology (∼11%) in this survey. This indicates that the Tat system is linked to bacteria that rely upon an anaerobic physiology. However, this finding also supports experiments in *E. coli* that show a constitutive expression of *tat* genes under both aerobic and anaerobic conditions [26].

Overall, almost all species that lacked a *tatC* homologue representing either Gram- negative or Gram- positive bacteria had reduced genome sizes ≤2 Mb and/or were obligate and opportunistic parasites which confirm findings from previous analysis [62]. The absence of *tatC* homologues within Gram- negative bacterial genomes appeared to be related to the size of the genome, as genomes ≤2 Mb typically lacked *tatC* sequences. For example, the obligate parasite phylum Mollicutes (n = 30 species; mean genome size 0.91 Mb) lacked *tatC* homologues as well as members from the thermophilic Thermotogae phylum (n = 14 species; mean genome size = 2 Mb). An exception was observed for obligate endosymbiotic α-proteobacterial members from Rickettsiales, with small average genome sizes of ≤1.3 Mb, yet all possessed a *tatC* homologue. Since many species within Rickettsiales are also known to be free-living, such as *Pelagibacter ubique* (SAR11), there may be a greater requirement for the Tat system in this expanding bacterial class. Gram- positive species that lacked recognizable *tatC* loci were generally opportunistic microflora of plants and animals, typically pathogens, likely due to the genome sequencing bias favouring pathogenic organisms. For instance, Bifidobacteriaceae of the phylum Actinobacteria, was the only Actinobacterial subgroup that lacked *tatC* homologues with the exception of *Bifidobacterium longum*.

The distribution of bacteria in possession of more than one *tatC* locus per genome was relatively narrow, where only 39 species from diverse phyla (corresponding to ∼5% of the total species surveyed) had at least two *tatC* loci as listed in Table 2. A previous analysis of TatC distribution [45] demonstrated that the occurrence of multiple *tatC* homologues was limited to Bacilli species, namely *Bacillus subtilis* and *Bacillus halodurans,* that are known to encode for two TatC isoforms, TatCd and TatCy. This *tatC* survey identified additional species with multiple *tatC* isoforms in the Bacilli class, *Bacillus*, in particular within *Paenibacillus*, *Lysinibacillus* and *Geobacillus* genera (Table 2). In fact, a few *Bacillus* and *Paenibacillus* species possessed up to four sets of *tatC* isoforms (*Paenibacillus sp.* Y412MC10, Table 2) indicating that functional diversification of these translocases is important for Bacilli and supported by experimental evidence for selective protein secretion in *Bacillus* [37], [40], [63], [64], [65]. Besides Bacilli species, the occurrence of multiple *tatC* loci/genome was very low (2%) and those in possession of more than one *tatC* locus typically shared a common environmental habitat (in particular those organisms living in soil and/or sea). Like Bacilli, the presence of multiple *tatC* loci/genome suggests that one or both of these additional Tat systems may support a specialized secretion function or alternatively protein secretion under a restrictive physiology as observed for Bacilli TatC isoforms. Hence, the presence of an additional Tat translocase system may allow a competitive advantage by supporting a specific protein secretion compared to other bacteria that possess a single TatC system in the same ecological niche.

Overall, our genomic survey of Bacterial genomes identified that 77% of all bacterial species possess one or more *tatC* loci and that only 5% have ≥2 *tatC* loci (Figure 1 and Table 2). The Tat translocase protein export system appears to be widely distributed throughout the Bacterial kingdom, and its absence may be associated with a symbiotic/endosymbiotic lifestyle since folded protein export is likely derived from the host. The occurrence of bacterial species with multiple *tatC* loci was low, but evidence suggests these additional Tat systems may be gained for specialized protein secretion as evident in the Bacilli class.

### Phylogenetic Analysis of Bacterial TatC Proteins

Phylogenetic analysis of 232 TatC proteins representing diverse taxa from all bacterial classes in possession of one or more *tatC* genes (including 20 species with >1 *tatC* locus) was performed to expand upon previous studies involving smaller datasets (∼70 sequences) [45] (Figure 2). Since a larger number of sequenced genomes were available at the time of this study, it was possible to evaluate the origins of single and multiple copy *tatC* members within bacteria, and to determine the origin of Bacilli TatC isoforms TatCd and TatCy. A chrenarchaeal *Vulcanisaeta distributa* TatC protein sequence served as the outgroup for this phylogenetic analysis as it represents one of the most ancient microorganisms that possesses a TatC homologue.

Our analysis outlined the division of the TatC protein members into four major clades, whose nodes are labeled as 1, 2, 3, 4 in Figure 2 and Figure S2. This analysis included TatC sequences from a diverse array of phyla in addition to previously examined Proteobacteria (α-, β- and γ- classes), Firmicutes and Chlorobia/Bacteroidetes, respectively. In general, TatC sequences clustered together in accordance to the 16S rDNA inheritance of their species within clades 1–3, which included phyla Proteobacteria (α-, β- and γ- classes), Firmicutes and Chlorobia/Bacteroidetes, respectively (Figure 2 and Figure S2); this finding is in agreement with previous findings [45], [62]. The monophyly of TatC homologues from Firmicutes within our phylogenetic tree, although supported by a weak posterior probability (PP) value of 0.5 (Figure S2), underpinned the hypothesis that Mollicutes, which apparently lack TatC, lost *tatC* and other genes as their genome reduced during evolution [66]. The lack of *tatC* genes in the genomes of other obligate intracellular parasites observed in our TatC distribution survey strongly supports this argument. Clades 1–3 also demonstrated evidence supporting internal gene duplication events, as many species in possession of more than one TatC in these clades branched together at the same nodes (Figure 2 and Figure S2). The only exception to this was Bacilli TatCd/y isoforms which formed a distinct grouping within clade node 2 (discussed in the next section).

Branching arrangements within the fourth clade at node 4 demonstrated a different clustering of TatC homologues from diverse taxonomic origins and included species from Actinobacteria, Cyanobacteria, Deinococcus-Thermus, Acidobacteria phyla, and from the class δ-proteobacteria (Figure 2 and Figure S2). The lack of linear inheritance within these branches suggests that these TatC sequences are likely undergoing greater rates of change that are not driven by the host genome. Bayesian analysis of the 232 protein dataset and other phylogenetic methods that included Neighbor Joining and Maximum Likelihood analyses all failed to resolve branches of clade 4 TatC sequences (PP value of 0.5). Upon closer inspection of their respective *tatC* operons, evidence of transposase/integrase genes and insertion sequences were identified at *tatC* loci of 15% of all clade 4 species (+/−8 genes from the *tatC* loci). This indicates that horizontal gene transfer influences clade 4 branching patterns in contradiction to previous findings [62], which is likely due to the large number of more taxonomically diverse bacterial genome sequences now available since the previous study. In fact, three major branches within clade 4, indicated as nodes 4a, 4b, 4c in Figure 2 (Figure S2), were enriched with taxonomically unrelated TatC sequences with reasonable confidence (PP≥0.7). This suggests that the environmental niche of the host in each of these branches may be driving TatC acquisition. For example, node 4a (PP = 0.7) showed clusters of TatC homologues from organisms commonly associated with soil dwelling organisms that grow on rotting materials, namely Actinomycetales (Actinobacterial order), Myxococcales (δ-proteobacterial order) and members of phyla Acidobacteria and Gemmatimonadetes. Node 4b showed an enrichment of aquatic/marine sediment dwelling species with TatC members from radiation tolerant Deinococci and Rubrobacterales, ammonium oxidizing Nitrospira, and sulphate reducing Thermodesulfobacteria. Node 4c was enriched with anaerobic methane utilizing species from gastrointestinal animal microbiota Coriobacteria (phylum Actinobacteria) and the class Verrucomicrobiae that reside in thermoacidic muds. A final example supporting an environmental bias on TatC variation was observed in the scattered branching patterns of δ-proteobacterial TatC sequences throughout the dendrogram, labeled in Figure 2 (and Figure S2) as δ1, δ2 and δ3 based on their taxonomic orders within this class.

Internal gene duplication events may explain the presence of multiple *tatC* loci in the genomes of eight species dispersed throughout the phylogenetic tree from γ-proteobacteria (*Colwellia psychrerythraea*), δ-proteobacteria (*Geobacter lovleyi*), Actinobacteria (*Xylanimonas cellulosilytica*, *Nakamurella multipartita*, *Streptomyces hygroscopicus*), Bacteroidetes (*Cytophaga hutchinsonii*), Acidobacteres (*Candidatus Solibacter usitatus*) and Nitrospira (*Candidatus Nitrospira defluvii*). Each homologous TatC pair from these 8 organisms grouped independently with PP≥0.9 supporting gene duplication events (Figure S2). Nonetheless, in our analysis there were also instances of multiple TatC homologues where one or more multiple TatC sequences shared a close association to unrelated taxa that reside in the same ecological niche (*i.e. Thermodesulfobium narugense*, *Sulfobacillus acidophilus*, labeled in Figure 2 and Figure S2 as *Tn* and *Sa*, respectively).

Phylogenetic analysis of the TatC protein family demonstrated evidence for combination of linear and environmental-driven inheritance, suggesting that evolutionary pressures acting upon TatC in free-living organisms could account for specialized Tat systems able to confer evolutionary advantages in environmental niches with strong ecological competition. The occurrence of multiple TatC homologues in single species apparently arisen from either intra-genomic events of gene duplication or horizontal gene transfer events further supports this hypothesis, thus potentially expanding the idea of the presence of functionally distinct Tat systems within single organisms, so far narrowed to Bacilli class [37], to a broader range of taxonomic groups.

### Bacilli TatCd and TatCy Form Two Distinct Clades

Since *tatC* loci found in Bacilli genomes appeared to be evolving separately from all other multiple TatC homologues, (Figure 2; Figure S2), a focused phylogenetic examination of Bacilli TatC sequences and their corresponding operons was performed and shown in Figure 3. The aim of this analysis was to clarify the origin of these multiple isoforms and determine the selective pressures that guide their potential paralogous evolution. In addition to phylogenetic analysis, an examination of each *tatC* locus and genes found within and adjacent to the respective locus was performed to provide insight into potential substrates for the translocase that may influence isoform divergence. To date, only *B. subtilis* TatCd and TatCy proteins are experimentally characterized isoforms of TatC where each isoform directs the secretion of particular Tat targeted substrates [38]. Phylogenetic analysis of Bacilli TatC proteins shown in Figure 3 demonstrate that among the 57 Bacilli TatC isoforms sequences identified in this study (listed in Table 2), 47 TatC sequence paralogs from the genus *Bacillus* clustered into two distinct clades that associated with either the characterized *B. subtilis* TatCd or TatCy. The division between the TatCd and the TatCy clade is marked by a triangle to indicate the point where the TatCd-TatCy split can be confidently placed (Figure 3). This includes bacilli species with 2 TatC homologues/genome such as *B. atropheus*, *B. amyloliquefaciens*, *B. licheniformis*, *B. megaterium*, *B. halodurans,* where doubled copies distributed into either the TatCd or TatCy branches suggesting that gene duplication events within TatCd and TatCy produced these clusters. Additionally, distantly related Bacilli species shown in distal branch nodes y4 to y7 suggest that TatCy is the progenitor of TatCd. It appears that transposition also plays a large role in the movement of TatCy members within Bacilli, as genes encoding transposons (IS605 family transposase *orfB*) and integrases (*xerC*) were identified in a 8 gene radius in either frame from *tatC* loci as shown during operon conservation analysis (Figure 3). Genomes of *Paenibacillus*, *Geobacillus* and *Exiguobacterium* appear to be the targets of *tatCy* transposition suggesting two scenarios for *tatCy* acquisition/origin: gain from a closely related Bacilli member, or gain from an unrelated bacterium in possession of a *tatC* operon found in its habitat.

Examination of the TatCd and TatCy clades in Figure 3, showed three main subclades (d1, d2, d3) and four within the TatCy clade (y1, y2, y3a, y3b, y4-y7). Overall, Bacilli species represented in each clade demonstrated branching patterns similar to their 16S rDNA relationship. After closer examination of *tatCd* operons and branching patterns, a trend emerged showing a relationship between phylogenetic clustering and gene conservation at the *tatC* operon region (Figure 3). *tatCy* operons showed a high frequency of association (71–82%) with genes associated with molybdenum cofactor biosynthesis proteins (*moaC*, *mogA*), with chaperones (*groE*), and 40% frequency of association to various vitamin/cytochrome and cofactor biosynthesis genes (Figure 3). As TatCy nodes became more distal (y4–y7), the strict gene conservation at the *tat* operon diminished. However, genes encoding for metal ion (Fe, Pb, Zn) transporters and cofactor biosynthesis, such as high-affinity Fe^2+^/Pb^2+^ permease (*FTR1/efeU*), iron binding lipoprotein/imelysin peptidase M75 (COG2822) and the known Tat substrate ferrous iron dependent DyP-type peroxidase (*efeB/ywdH*) [43], [64] were present in 55% of the *tatC* operons examined in these nodes. Previous experiments have shown that the secretion *efeB* is effected by the environmental salinity of the growth medium [40]. Other potential Tat dependent candidates were located less frequently (20–40%) adjacent to or as part of a *tatCy* operon at nodes y5–y7, such as thioredoxin reductases (*trx/ydbP*) [67] and various oxidoreductases [68], [69], [70] (data not shown). Additonally, genes found in *E. coli tatC* operons, specifically ubiquinone oxidoreductases and methyltransferases were also found at a frequency of 5% and 18% respectively in Bacilli *tatC* loci.

*tatCd* operon analysis at branch node d1 in this sub-branch showed high gene associations to the Tat substrate phosphodiesterase D (*phoD*) gene that confirms previous findings [37] but also identified pyrrolidone-carboxylate peptidase (*pcp*), a periplasmic protein involved in pyroglutamyl signal sequence cleavage [71]. PhoD secretion was previously demonstrated to be a *B. subtilis* TatCd dependent process and its secretion is influenced by phosphate availability [72]. The remaining TatCd sequences from other Bacilli species at branch nodes d2 and d3 all lacked the *phoD* gene in the *tat* operon and in its place had conserved genes with as yet undefined functions (DUF839; DUF2535). Additionally, genes encoding proteins involving a bound fatty acid like DegV domains (*degV*) and dihydrofolate reductase (*dfrA/DHFR*) known to be tightly folded protein in the cytoplasm [73] were also frequently found (60–70%) in *tatC* operons surveyed in nodes d2–d3 (Figure 3). It is important to note that recent transcript profiling analysis of *B. subtilis* has shown that *tatCy* is expressed under 104 different culturing conditions while *tatCd* of *B. subtilis* was only expressed under phosphate limitation [74] indicating the specialization of TatCd function.

In summary, Bacilli TatC phylogenetic analysis demonstrated that TatCd and TatCy form two distinct clades, where TatCy appears to be the progenitor of TatCd. Operon gene mapping has identified an assortment of candidates that frequently associate with *tatC* genes in addition to known Tat targeted substrates. Many of these candidate genes are related to metal cofactor utilization/biosynthesis. This operon analysis has identified known Tat substrates and may also provide insight into an alternative Tat substrate candidate identification method in addition to twin arginine leader sequence prediction software.

### Mean dS/dN Analyses Identify Bacterial Classes with Different *tatC* Selective Pressures

The ratio of synonymous (dS) to non-synonymous (dN) nucleotide substitution was calculated for all pairwise comparisons of gene sequences coding for 232 taxonomically representative TatC homologues. The purpose was to determine which bacterial classes possessing *tatC* sequences were undergoing diversification through positive selection (dS/dN <1) in comparison to those classes with higher *tatC* conservation (dS/dN >1) that indicated sequences that are actively maintained in the host genome. Overall, the mean dS/dN value of all pairwise comparisons of *tatC* homologues was 2.62, indicating that bacterial *tatC* sequences are maintained under selective pressure, albeit much lower than initially expected. This result reflected a high proportion of non-synonymous sequence variation occurring in different bacterial classes and dS/dN values for species from various bacterial classes ranged from 0.35 to 5.99 (Table S2).

Our analysis of dS/dN values from 23 bacterial *tatC* possessing classes showed that five classes including Actinobacteria and Deinococci had the lowest median dS/dN values (1.0–2.0) in comparison to γ- proteobacteria, ε- proteobacteria, Bacteroidetes and Bacilli with the highest median dS/dN (3.0–4.0) values (Figure 4). Low median dS/dN values for Actinobacterial and Deinococcal species indicated that *tatC* sequences have undergone extensive sequence variation in comparison to other classes. This can be interpreted as *tatC* divergence towards a specialized function or towards the lack of selective pressures to maintain functional *tatC* copies in these hosts that no longer require translocase activity. Alternatively, low dS/dN values may reflect higher rates of unrelated horizontally transferred *tatC* sequences, as codon usage will vary depending on the origin of the transferred *tatC* sequence. This outcome also supports our TatC phylogenetic analysis, as many TatC members within these classes clustered together with taxonomically unrelated species according to a common environmental pressure (clade 4; Figure 2 and Figure S2).

The majority of bacterial *tatC* classes examined (52% of 23 classes) resided within the intermediate median dS/dN value range (2.1–3.1) as shown in Figure 4. In general, these intermediate dS/dN *tatC* homologues represented a diverse group largely composed of Gram- negative organisms and a single Gram- positive class (Clostridia). The intermediate median dS/dN values for these bacterial classes indicated that the overall mean dS/dN value of 2.62 accurately reflected the majority of bacterial class *tatC* sequence variation in bacteria. The broadest ranges of dS/dN values among bacterial classes in this group were found in within Verrucomicrobia (2.74–3.76), β-proteobacteria (1.62–5.10) and δ- proteobacteria (2.10–3.36), strongly suggesting that *tatC* homologues in these classes have significantly different selective pressures directing sequence conservation. High *tatC* dS/dN values observed for β-proteobacteria and δ-proteobacteria were particularly interesting, since high dS/dN values generally corresponded to species that possessed more than one *tatC* locus/genome (Table 2). For example, β-proteobacterial species with the highest *tatC* dS/dN values (>4.45) were related to *tatC* homologues from *Methylobacillus flagellatus* and *Methylotenera mobilis*, suggesting that multiple *tatC* copies may have greater pressures to maintain synonymous conservation to support conditionally dependent Tat system substrate secretion activity in these methylotrophic organisms.

The highest median dS/dN values (>3.1) were observed for γ- proteobacteria, ε- proteobacteria, Bacteroidetes, and Bacilli and indicated that *tatC* homologues in these classes were maintained under the highest selection pressure. Bacterial classes with high median *tatC* dS/dN values also tended to possess more than one *tatC* locus/genome, as observed for multiple *tatC* sequences found in species from Bacilli and γ- proteobacteria (Table 2). As observed for β- proteobacteria and δ- proteobacteria with multiple *tatC* copies, we also noted higher dS/dN values (2.9–4.5) for Bacilli and γ- proteobacterial species with more than one *tatC* copy suggesting that multiple *tatC* copies have greater selective pressures acting upon them. As dS/dN values increased in bacterial classes we surveyed, the frequency of identifying multiple *tatC* copies also increased (Table 2). However, multiple *tatC* found in Actinobacteria (lowest dS/dN values) proved to be an exception to this trend and supports a role for lateral gene exchange in common niches influencing high dS/dN values.

Examination of dS/dN analysis of bacterial *tatC* homologues from various classes, revealed that the selective pressures acting upon *tatC* sequence variation support our phylogenetic analyses, which demonstrates that TatC evolution is largely directed by the taxonomic origins of its host but also appears to be driven by its environmental pressure. Multiple *tatC* loci present in bacterial classes also tend to be maintained at higher dS/dN (with the notable exception of Actinobacteria), suggesting that *tatC* duplicates fulfill important functional purposes for their host.

### Bacterial TatC Amino Acid Conservation

Analysis of individual TatC amino acid and nucleotide conservation from multiple alignments was performed to identify any conserved residues within TatC and identify other conserved residues involved in substrate binding regions and/or TatC multimerization contacts. This was carried out by calculating the extent of conservation from aligned amino acids and the extent of synonymous substitutions in each corresponding codon sequence in the nucleotide codon alignment. The most conserved amino acid according to its percentage identity (% identity) and its associated mean synonymous nucleotide substitution (Sd) provide an estimation of the selective pressure acting on particular residues and domains within the protein. The TatC protein alignment used for phylogenetic analyses and its corresponding codon aligned nucleotide sequences were used for these analyses. This resulted in an amino acid sequence where the highest % identity was reported for each residue in alignment (the consensus sequence) in addition to its respective mean Sd value for each codon.

The mean synonymous substitution (Sd) values identified many regions undergoing variable rates of conservation throughout the bacterial TatC protein (Figure 5). Our analysis of 232 TatC sequences demonstrated that residues were conserved at greater extent within the amino- terminus and first two loops of TatC rather than in TM1 strand. Residue conservation increased in frequency, particularly within TM2–TM5 regions, in the carboxyl-terminus of the TatC consensus protein (Figure 5). This indicated the importance of these TatC TM residues and almost all are proposed to participate in substrate leader peptide binding pocket described for the *A. aeolicus* TatC structure [27] and as shown in the *E. coli* model of TatC (Figure 6). Examination of mean Sd values at all codon positions of aligned *tatC* sequences (grey chart in Figure 5), indicated that codon conservation was analogous to estimated amino acid conservation except for three residues found in TM regions, TM2 (W85), TM3 (Y119) and TM4 (F162) specifically. These exceptions suggest that these codons may be more likely to undergo non-synonymous nucleotide substitutions, possibly to amino acids with similar chemical characteristics. In *E. coli* TatC, only TM3 Y126A (TatC consensus Y119) was shown to be important for TatC structure and function based on site-directed mutagenesis experiments (Table 3) [29], [30], [36]. These three Sd values exceptions may reflect significant differences in hydrophobic amino acid codon usage in TM regions between the diverse bacterial phyla surveyed in this study or possibly reflect TM strand residue variation in the various α- helical membrane segments.

**Table 3:** Summary of *E. coli* TatC amino acid variants experimentally determined to alter Tat translocase activity and residues known to interact with twin arginine leader peptides.

TatC consensusamino acid residueand number inthis study	Location withinN- or C-terminus TMor loop (L)	*E.coli* TatC aminoacid consensusposition	Genetically selectedtranslocation inactivating *E. coli*variants [31]	*E. coli* Ala substitutionvariants that reducedtransport function[29], [30], [36]	Residues capableof cross- linkingto TorA twinarginine leader peptide[34]	Residues capable ofphoto-affinity labelling withTatA, TatB, or TatC protein[34]
NA	N	V3			Yes – high	TatB
NA	N	E4			Yes	
NA	N	D5			Yes	
NA	N	L9			Yes – high	
I1	N	I10			Yes	TatB
H2	TM1	H12		Yes		
E5	TM1	I14				TatB
E6	TM1	E15		Yes	Yes– high	
L7	TM1	L16			Yes	
R8	TM1	R17		Yes		
A37	L1	S46	S46F			
P39	L1	P48	P48L, P48S	Yes		
L52	L1	M59	M59K			
I53	L1	I60	I60N			
G56	L1	D63	D63V			TatC
V57	L1	V64	V64E			
E59	L1	S66	S66P			
F61	L1	F68	F68S			
T63	L1	T70	T70R			
V67	TM2	L74	L74P			
F87	TM2	F94	F94S			
P90	TM2	P97		Yes		
L92	L2	L99	L99P, L99Q			
Y93	L2	Y100		Yes	Yes	
K94	L2	K101			Yes	
E96	L2	E103	E103G, E103K	Yes		
Y119	TM3	Y126		Yes		
P124	TM3	P131	P131L			
L130	TM3	L137	L137H			
V138	L3	V145	V145E			
M142	L3	S148	S148P			
D144	L3	D150	D150G, D150V			TatA, TatB
I145	L3	I151	I151T			
E164	TM4	E170		Yes		
E181	L4	E187			Yes	
A199	TM5	M205				TatB*,TatC*
D205	TM5	D211		Yes		TatA, TatB**
Q209	L5	Q215	Q215R	Yes		
L219	TM6	L225	L225P			
E221	TM6	E227			Yes	

*ND*, no difference from wild-type TatC; *NA*, not alignable in this study.

* *E. coli* TatB L9C variant formed Cys cross link to TatC and interaction observed between TatC M205R to TatB F2L or F6L variants [31].

** Experimentally shown to destabilise TatB-TatC interactions [30].

Our analysis of TatC residue conservation identified a total of eleven highly conserved (≥75% identity) amino acid sites from TM3 to the carboxyl terminus of TatC (highlighted in grey boxes in Figure 5) that have not been previously examined (Table 3). These eleven TatC consensus residues reside in TM3 (L110, F111, F129), TM4 (F159, G160, F162, P165), loop 4 (L183, K187) and TM6 (P216, E221) regions of the protein and may provide additional insights into TatC function when studied biochemically. The other highly conserved residues in Figure 5 have been studied yet were shown not to significantly alter *E.coli* TatC translocation function when replaced with either Ala [29], [30], [36] or with a variety of amino acids with opposite characteristics [31].

To highlight the pattern of overall TatC residue conservation and variability, highly conserved (≥75% identity) and poorly conserved (≤25% identity) residues were mapped onto a model structure of *E. coli* TatC shown in Figure 6. This analysis revealed a striking pattern of conserved and variable regions in the three dimensional structure. As observed from secondary structure analysis of TatC proteins provided in Figure 5, periplasmic facing TatC loops (loops L2, L4) demonstrated the highest abundance of variable residues (highlighted red residues). These periplasmic regions appear to be linked to interactions with other Tat system components, TatA and TatB, and all of these positions residues had moderate to low conservation suggesting location or structural features may be of greater importance than residue property (Table 3) [34]. Previous site directed mutagenesis experiments of TatC have reported that the substitution of many moderate to poorly conserved residues in *E. coli* TatC resulted in functional inactivation of translocase secretion [31]. Such residues, as shown in Figure 5, resulted in low Sd and % identity values and may represent regions undergoing more frequent alterations to accommodate different Tat translocase components. Conversely, the cytoplasmic facing loops had the highest abundance of conserved residues. A face of each TM strand end, with the exception of TM6, also had an abundance of conserved residues (Figure 6). The proposed substrate TatA interaction pocket of TatC [27] also had a diagonal region of highly conserved residues that began from its cytoplasmic amino- terminus at TM1 that increased towards the periplasmic regions of TM5 and a small portion of TM6 (Figure 6). The majority of the eleven conserved TatC residues identified from TM3, TM4, TM5 in the consensus alignment were found in this pocket region of the model suggesting their involvement as part of binding pocket and/or structural support in this domain.

### Bacterial TatC Consensus Sequences Comparisons to Bacilli TatCd/TatCy Isoform Sequences

**Figure 7:**
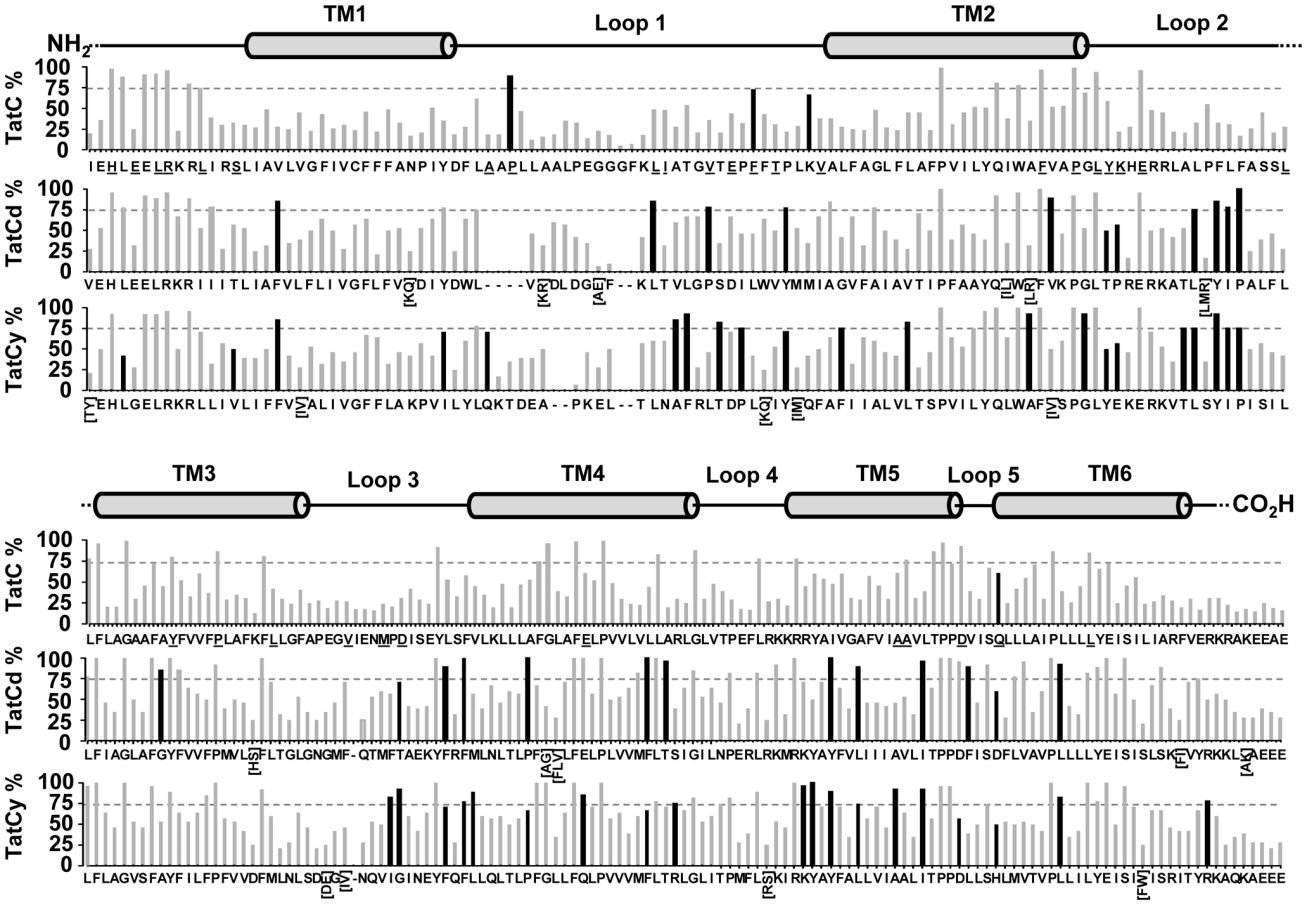
Amino acid conservation alignment between the overall bacterial TatC consensus sequence and bacilli TatCd and TatCy consensus sequences. The extent of residue conservation (% identity) is shown on each y-axis as bars and indicates the extent of amino acid conservation calculated at each position within the 232 TatC consensus alignment (TatC % identity; top bar chart), the bacilli TatCd alignment of 31 sequences (TatCd % identity; middle bar chart), and a bacilli TatCy alignment of 26 sequences (TatCy % identity; bottom bar chart). The single letters listed on the x-axes of each bar chart represents the most conserved amino acid according to its percentage identity (y-axes) for TatC (top chart), TatCd (middle) and TatCy (bottom) for all alignable positions from amino (NH_2_) to carboxyl (CO_2_H) terminus. The extent of amino acid conservation (% identity) is shown on the y-axis as bars, where grey bars indicate percentage identity values determined for all TatC consensus residue positions, while black bars highlight significant changes between amino acid residue positions of bacilli TatCd/TatCy compared to the total TatC consensus. Regions of secondary structure are represented as a cartoon diagram where protein loops and termini are shown as lines and predicted TM α- helix domains (grey cylinders) and are aligned according to the total TatC amino acid consensus.

The final objective of this study was to compare the extent of amino acid conservation within Bacilli TatCd and TatCy isoforms to the bacterial TatC consensus to identify regions or residues in each TatC isoform that may contribute to specialized substrate secretion in Bacilli. Since Bacilli *tat* operons lack *tatB*, this analysis was also performed to determine residues that interacted with TatA and TatC specifically, and identify conserved residues specific for only TatA-TatC associations. A comparative TatC-TatCy/Cd conservation (according to % identity) consensus alignment is provided for each amino acid position from amino- to carboxyl- terminus in Figure 7.

In general, highly conserved residues identified in the overall bacterial TatC consensus were generally similar to those identified in Bacilli TatCd and TatCy, and the majority of variation from the main consensus was observed sporadically after loop1 throughout the carboxyl terminus of the protein (Figure 7). Notable variations between the bacterial TatC consensus and TatCd/Cy consensus sequences were also observed for consensus aligned residue L4 (amino- terminus) as this position was substituted with an amino acid with similar R-group property in Bacilli TatCd and TatCy. Variations in the loop 2 YK portion of the P^91^GLYK^94^ motif in both Bacilli TatCd (TP^94^) and TatCy (YE^100^) suggest that these residues might contribute to signal sequence specificity since these regions were shown to crosslink to the twin arginine leader peptide in *E. coli* experiments [34]. Other known twin arginine leader peptide interacting residues E181 in loop 4 and E221 in TatC TM6 (Table 3) were only moderately conserved in their respective positions in Bacilli TatCd (E180 and E220) and TatCy (M187 and E227). Therefore, substrate signal peptide binding regions were typically maintained in Bacilli TatCd and TatCy isoforms with the notable exception in loop2.

The conservation of amino acid residue positions known to contact TatC-TatB based on *E. coli* TatC photoaffinity crosslinking experiments [34] were difficult to distinguish from TatA sites since many of these positions were not highly conserved in the amino- terminus of the bacterial TatC consensus (Figure 5) nor in bacilli TatCy and TatCd consensus (Figure 7). Since almost half of the bacterial classes surveyed lacked *tatB*, our bacterial TatC alignment would be expected to see moderate conservation of TatB-TatC interacting residues (Table 1). Bacterial TatC consensus positions I1 and E5 (Table 3) that were previously shown to contact TatB in *E. coli* TatC photoaffinity labelling experiments [34] appear to be maintained under low to moderate conservation in both the TatC consensus as well as in Bacilli TatCd (V1 and E5) and TatCy (T/Y1 and G5) consensus residues (Figure 7). It appears that these contacts were not conserved in either Bacilli TatCd or TatCy amino-terminus, and not within the overall alignment of bacterial TatC, suggesting that location rather than residue property may be important in TatB-TatC interactions. In contrast, TatB and TatA contacting residue D144 (Table 3) located in loop3, showed that alternative residues were highly conserved (>70% identity) in each Bacilli isoform, TatCd (T143) and TatCy (G150). This variation in periplasmic loop3 may suggest that TatAd and TatAy differences may influence residue conservation at this position in either protein (since TatB is absent in Bacilli) and are worthy of further experimental characterization. Additionally, *E. coli* TatC genetic inactivation experiments substituting D150G which resulted in transport inactivating phenotypes [31], showed that glycine is favoured (% identity) in Bacilli TatCy consensus at this position (Figure 7). Since Bacilli TatCy translocation activity has been characterized experimentally [38], it would be interesting to determine if substitutions introduced into *E. coli* TatC residues result in the same phenotype in each of the Bacilli TatC isoforms. If *E. coli* TatC D150G is important for function, the outcome may be determined since the overproduction of *B. subtilis* TatCd was shown to aid in the secretion of YwbN, which relies on the TatCyAy system [40]. Finally, the TatB-TatC specific contact position, A199 in TM4 and the TatA-TatC specific contact position D205 in loop5 (Table 3) demonstrated moderate to high conservation (% identity) respectively when comparing the overall TatC consensus to Bacilli TatCd/y consensus sequences. Higher % identity values for D205 compared to A199 in Bacilli TatCd and TatCy stress the specificity of interaction of these residues with TatA and TatB respectively and underline These periplasmic regions appear to be linked to interactions with other Tat system components, TatA and TatB the importance to maintain D205 rather than A199 in Bacilli.

Overall, the bacterial TatC consensus and Bacilli TatC isoform consensus alignments have identified potential amino acid positions that may serve as additional targets for future analysis of TatC sequences. Since roughly half of all bacterial classes lack *tatB* genes, this comparative analysis of bacterial versus Bacilli TatC isoform consensus residues highlighted in Figures 5 and 7 may be useful in identifying useful interaction domains but also in determining the role of TatB in the translocase system.

## Conclusion

The analysis performed on bacterial TatC proteins has provided new insights into the distribution of TatC protein family and organization within *tat* operons from various bacterial classes (Figure 1 and Table 1). Bacterial TatC distribution in this survey confirms that the majority of bacteria possess at least one minimal Tat system (TatA and TatC) after the surveying genomic *tatC* loci. Bacteria lacking a *tatC* gene were generally bacteria with smaller genomes of less than 2 Mb as well as microorganisms that adopt an obligate intracellular lifestyle.

Our analysis of the bacterial TatC phylogeny has determined that TatC is evolving in two directions, the most common being inheritance and while the second direction is attributed to horizontal gene transfer between unrelated bacteria residing in a similar environment. Phylogenetic analysis of TatC proteins confirmed that the majority of bacterial clades (3 out of 4) followed taxonomic inheritance in agreement with previous findings [45], but has now identified a smaller fourth clade comprising unrelated bacterial taxa that share common environmental niches (Figures 2 and 3). This is important because it adds a new dimension to consider for Tat system inheritance. If Tat system inheritance can be rapidly gained between unrelated organisms, it may increase the virulence of previously non- pathogenic microorganisms and/or alter bacterial ecosystems by improving the fitness of previously poor competitors within that niche. Phylogenetic analysis of Bacilli TatC isoforms determined that TatCy is the progenitor of TatCd (Figure 3), suggesting that TatCy may be more representative of single TatC homologues present in other species. Bacilli TatCy homologues were associated with nearby (+/−8 genes from the *tatC* locus) with transposon/integron gene elements compared to TatCd. This suggests that Bacilli TatCd have undergone rapid diversification from their originally duplicated TatCy sources. The combination of Bacilli TatC phylogenetic analysis with *tatC* operon mapping, also revealed an assortment of interesting gene associations that may be potential substrates/or linked to pathways specific for TatCd/y isoform substrates (Figure 3). This analysis provided a list of candidate Tat substrates and biosynthetic pathways that involve one or both Bacilli TatC isoforms helping determine the directions of TatC isoform specialization. In particular, *tatCd* and *tatCy* operon analyses identified the presence of known TatCd or TatCy substrate genes, such as *phoD* and *ywbN*, as part of, or close to, *tatC* loci lending support and confidence that other closely associated genes to *tatC* may also be potential Tat substrate candidates (Figure 3). It is also important to note here, that experiments involving Tat substrates lacking one of the two arginines in its twin arginine leader sequence may still be recognized and secreted by the Tat system [75]. The loss of both arginines from leader peptide result in a lack of secretion unless the first cytosolic domain of TatC sustains variations to compensate [76]. This suggests that TatC diversification towards specialization may complicate Tat substrate identification by leader predicting algorithms and validates operon mapping strategies used herein as an additional identification method.

Examination of synonymous to non- synonymous substitution rates (dS/dN) within diverse *tatC* encoding taxa determined that *tatC* sequences were more variable sequence in Actinobacteria and Deinococci while γ- proteobacteria, ε- proteobacteria, Bacteroidetes and Bacilli were maintained under the highest rates of conservation (Figure 4). This shows that TatC sequence conservation is much more variable than would be expected for an important secretion system. Our study has also identified *tatC* homologues from diverse bacterial classes that merit further experimental characterization. Beyond evolutionary considerations, the approaches used herein combining synonymous/non-synonymous distributions and amino acid conservation from alignments, have identified 11 conserved uncharacterized residues and many variable residue positions within the overall bacterial TatC consensus alignment that can assist further studies of this fascinating translocation system (Figures 5–7).

## Supporting Information

Figure S1 A protein alignment of all 233 TatC proteins examined in this study. Amino acids for each of the 233 TatC proteins are provided and show all alignable positions examined in this study. Abbreviated TatC sequence tags are provided for clarity and correspond to bacterial genus and species names provided in Additional file 1. Regions highlighted in blue have been predicted to be transmembrane strand regions. The degree of blue highlighting behind each amino acid indicates how many transmembrane prediction programs have assigned that amino acid to a transmembrane segment (three to one according to the shift from the darkest to the lightest blue) Yellow and black bar charts shown at the bottom x-axis of the amino acid alignment indicate the amino acid conservation (top yellow chart) where bar height is proportional to the conservation of physico-chemical properties for each column of the alignment, quality (middle yellow chart) where bar height and shading is proportional to the number of alignable residues and % amino acid identity at each position and the amino acid consensus (bottom black chart) representing the most frequently identified amino acid for each alignable position.
(PDF)

Figure S2 A rooted phylogenetic tree of bacterial TatC proteins. The rooted dendrogram was generated by Bayesian analysis of 233 TatC sequences from diverse bacterial taxa where the archaeal *Vulcanisaeta distributa* TatC sequence (labelled as Vdistrib01, GenBank: YP_003902595.1) served as the outgroup for this analysis. Symbols adjacent to taxa indicates multiple TatC copies from the same genomes discussed in the Results and Discussion section and the corresponding species are detailed as follows: *Cytophaga hutchinsonii* (•); *Geobacter lovely* (••); *Colwellia psychrerythraea* (•••); *Xylanimonas cellulosilytica* (x); *Streptomyces hygroscopicus* (xx); *Candidatus Solibacter usitatus* (xxx); *Nakamurella multipartita* (¥); *Candidatus Nitrospira defluvii* (¥¥); *Sulfobacillus acidophilus* (Sa); *Thermodesulfobium narugense* (Tn); *Thermodesulfatator indicus* (Ti). Numbering in light blue beneath each node represents posterior probability (PP) confidence estimates where 1 represents branching patterns with the highest confidence values and 0 no confidence in relationship at the node. Only PP values equal or greater than 0.6 are shown. Nodes with large black numbers highlight important clade divisions (1–4) between branches and Greek delta (d) symbols written beside these clade numbers indicate important delta proteobacterial divisions within that numbered clade. Bacterial classes enriched in major clusters are highlighted on the right hand side of the dendrogram, and in the case of Bacilli TatC sequences, TatCd and TatCy isoforms are also indicated.
(PDF)

Table S1 A complete list of all 1415 bacterial *tatC* genome sequences obtained from NCBI for this study.
(XLS)

Table S2 The distribution of mean synonymous to non-synonymous (dS/dN) substitutions within *tatC* sequences from various bacterial classes.
(XLS)
