# Supplementary figures and images for: Diversity and Evolution of Bacterial Twin Arginine Translocase Protein, TatC, Reveals a Protein Secretion System That Is Evolving to Fit Its Environmental Niche
Source: PLoS One. 2013 Nov 13;8(11):e78742. doi: 10.1371/journal.pone.0078742 (PMC3827258; doi:10.1371/journal.pone.0078742)

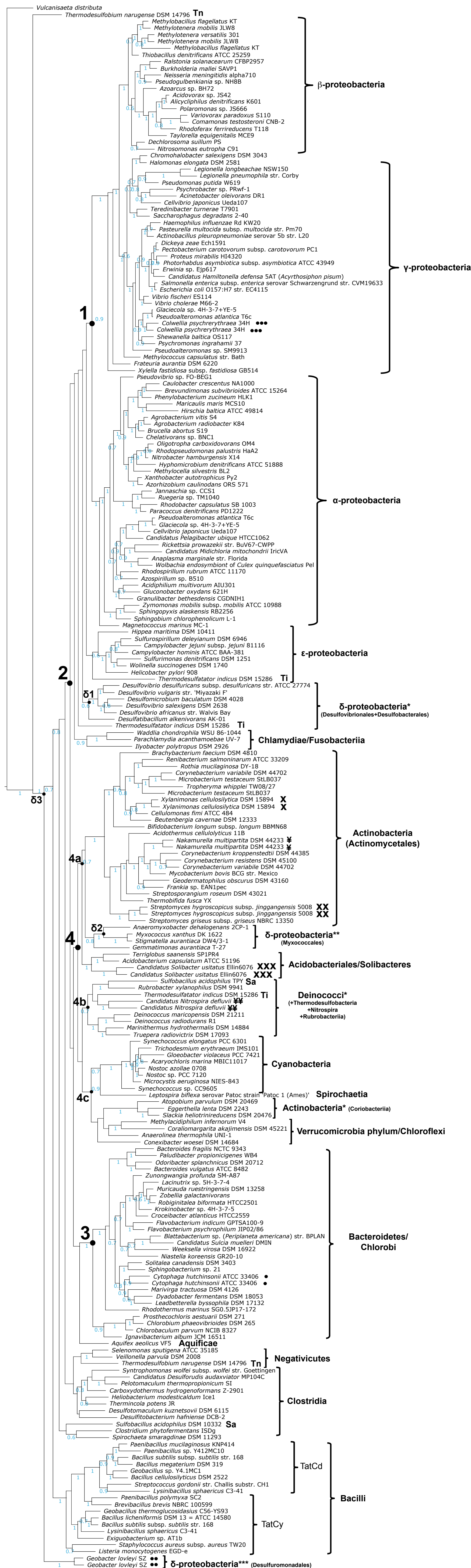

Supplement: Figure S2 — A rooted phylogenetic tree of bacterial TatC proteins. The rooted dendrogram was generated by Bayesian analysis of 233 TatC sequences from diverse bacterial taxa where the archaeal Vulcanisaeta distributa TatC sequence (labelled as Vdistrib01, GenBank: YP_003902595.1) served as the outgroup for this analysis. Symbols adjacent to taxa indicates multiple TatC copies from the same genomes discussed in the Results and Discussion section and the corresponding species are detailed as follows: Cytophaga hutchinsonii (•); Geobacter lovely (••); Colwellia psychrerythraea (•••); Xylanimonas cellulosilytica (x); Streptomyces hygroscopicus (xx); Candidatus Solibacter usitatus (xxx); Nakamurella multipartita (¥); Candidatus Nitrospira defluvii (¥¥); Sulfobacillus acidophilus (Sa); Thermodesulfobium narugense (Tn); Thermodesulfatator indicus (Ti). Numbering in light blue beneath each node represents posterior probability (PP) confidence estimates where 1 represents branching patterns with the highest confidence values and 0 no confidence in relationship at the node. Only PP values equal or greater than 0.6 are shown. Nodes with large black numbers highlight important clade divisions (1–4) between branches and Greek delta (d) symbols written beside these clade numbers indicate important delta proteobacterial divisions within that numbered clade. Bacterial classes enriched in major clusters are highlighted on the right hand side of the dendrogram, and in the case of Bacilli TatC sequences, TatCd and TatCy isoforms are also indicated. (PDF) [file pone.0078742.s002.pdf]
